# Some Affections of the Eye Associated with and Dependent upon the Scrofulous Diathesis1Read before the Buffalo Academy of Medicine, February, 1894, and the New York State Medical Association, Fourth District Branch, at Its meeting Held in Buffalo, May 8, 1894.

**Published:** 1894-09

**Authors:** Alvin A. Hubbell

**Affiliations:** Professor of Ophthalmology and Otology, Medical Department of Niagara University, Buffalo, N. Y.; 212 Franklin Street


### SOME AFFECTIONS OF THE EYE ASSOCIATED WITH AND DEPENDENT UPON THE SCROFULOUS DIATHESIS.[1]

By ALVIN A. HUBBELL, M. D.,
Professor of Ophthalmology and Otology, Medical Department of Niagara University, Buffalo, N. Y.

The theme as here stated involves, according to present pathological conclusions, an erroneous assumption. It presupposes the existence of a condition of the human system heretofore defined as scrofula, whereas scrofula is known no more in the newer pathology. It has figured most prominently in the past; but its foundation has been narrowing more and more, till within the last few years it has been removed entirely. Those lymphatic enlargements and other conditions which were formerly regarded as scrofula are now known to be forms of tuberculosis. Today scrofula is tuberculosis. And without scrofula there can be no "scrofulous diathesis." But in losing a name we do not lose a disease or its predisposing conditions. The "thing" is still with us, but has only been newly labeled.

There may be some objection to the word "diathesis." Indeed, there are some who deny such a condition. Perhaps "vulnerability" or "tissue-vulnerability" is a term more in harmony with our present knowledge. But I like the word "diathesis," signifying to me, as it does, a condition, either congenital or acquired, in which there is found a weakened vital or cell-resistance to morbific agents, and, therefore, in a sense, a predisposition to some particu-

---

1. Read before the Buffalo Academy of Medicine, February, 1894, and the New York State Medical Association, Fourth District Branch, at its meeting held in Buffalo, May 8, 1894.

lar constitutional or local disease. It seems to me very expressive to speak of the "gouty" diathesis, "scorbutic" diathesis, "hemorrhagic" diathesis, and the like. For the present purpose, therefore, I will retain the old name, but with its modified signification.

The so-called scrofulous or strumous diathesis is peculiar, and is, generally speaking, easily recognized. It exhibits a proneness to inflammations of the skin and mucous membranes, especially inflammations of a chronic nature, and a susceptibility to tubercular infections leading to glandular enlargements, bone and joint disease, "cold abscesses," pulmonary phthisis, and similar affections. This diathesis might with propriety be called tuberculous, as it offers most fertile soil for the propagation of the tubercle bacillus.

It is a condition both inherited and acquired. It is seen in children of tuberculous parents, or of ill-nourished, over-worked or greatly debilitated parents. It may be acquired from insufficient or improper food, impure air, insufficient light, uncleanliness and unhealthy surroundings. It is peculiarly a condition of young life, and is found alike in both sexes. It may be seen, generally speaking, in two types of individuals, one of which possesses the so-called "sanguine temperament," the other the "phlegmatic" (Treves on Scrofula). The one type is also said to be erethistic, the other torpid (Keen's American Text-book of Surgery). In 1881, Dr. Mahomed and Mr. Galton exhibited, at the museum of the International Medical Congress held in London, a series of "composite" photographs which admirably illustrated the general physiognomy of these two types as found in phthisical patients. In the first, "the face is oval, the lower jaw small, the features delicate and regular, the lips thin, the eyes are bright and covered with long eyelashes." In the second type, "the face is broad, the lower jaw large, the smaller bones often prominent, the features coarse and irregular. The nose is generally thick, the lips tumid, the lobes of the ears large and the neck unshapely. Individuals of the first type "are tall, slight and graceful, with well-formed limbs, hands and feet, a fine clear skin, usually a fair complexion, and the hair is often remarkably fine and silken." The disposition is sprightly and excitable. Those of the second type are, as a rule, short and burly, with coarse limbs, large hands and feet. The skin is coarse, harsh and thick. The amount of subcutaneous cellular tissue is considerable. . . . Persons of this class appear flabby and heavy-looking; they are apathetic, have little muscular power and are soon tired." (Treves on Scrofula.)

Sir Richard Barwell (Heath's Dictionary of Surgery, Vol. II., page 297,) also recognizes two types of this diathesis, the one corresponding, he says—

To fineness and paucity, the other to coarseness and abundance of connective tissue. The former is marked by refinement and definition of the features. The curves of the lips, the nasal, aural and tarsal cartilages are finely modeled. The conjunctiva and sclerotic are so thin that the pigment of the choroid, partially seen through them imparts a bluish or pearl-grey color to the white of the eye; the skin clear and pure of tint, with cool-toned, ash-grey shadows, is so translucent that the bluish wavy course of subjacent veins is plainly marked, as on the upper eyelid, the temple, the angle of the jaw, etc.; the luscious redness of the lips testifies to the same condition, the eyelashes are abundant and a fine down, often rather long, extends from the margin of the scalp-hair some way down the forehead, the temples and nape. The whole aspect is of refined but fragile beauty.

The other type is coarse and ugly. The head large and lumpy, bigger before than behind, is flanked by large, red, puffy ears; the jaws are prominent and lips thick, ill-defined, and often cracked, sway clumsily apart. The nose is lumpish, the origin of the alæ ill-defined, the eyelids thick and clumsy, often, when not inflamed, bordered with red, and are frequently lined with dried Meibomian secretion clinging to the roots of the sparse, irregular lashes. The dull, unclean-looking skin is marked by large orifices of sebaceous glands. The figure is unusually ungainly, the joints and extremities large, the belly prominent; the hair coarse, either of a dull, sandy color or lusterless black.

This somewhat rhetorical description applies to the two extreme types. Between these there are various mixtures and combinations. Whatever description, however, may be given to this condition, one thing is certain: the vital resistance of tissues is weakened, especially that of the skin and mucous membranes, and the lymphatic circulation is sluggish. Thus morbific agents find lodgement, and with insufficient antagonism become operative, producing chronic inflammations of the skin and mucous membranes, especially of those mucous membranes most exposed to the air—lupus; eczema; tuberculosis of the glands of the neck, mesentery, joints, bones and lungs, and other similar diseases. Such subjects are particularly prone to naso-pharyngeal, laryngeal and bronchial catarrhs, to enlarged tonsils, adenoid vegetations, hypertrophies over the turbinated bones and to inflammations of the lachrymal passages and conjunctiva.

That this diathesis is an important factor in disease not to be lost sight of by the physician is further proved, not only by the prevalence, but by the fatality, of those diseases which are engrafted upon it. Not that every person who has catarrh of the nose or throat, or inflammatory affections of the eyes, or tuberculosis in some of its manifold forms was originally "diathetic," as here described, but that very many such were.

The affections of the eye which are liable to develop upon this tissue-state or diathesis are eczema of the lids, inflammation of the margin of the lids, some forms of inflammation of the conjunctiva and cornea, inflammation of the lachrymal sac and of the iris and choroid, lupus of the lids and conjunctiva, and tubercular disease of nearly every structure of the eyeball and its appendages. In this discussion but little detailed consideration need be given to lupus and other tuberculous affections, as they are rare. The inflammatory affections are most frequent and will engage our attention at this time.

It is generally accepted today that all inflammations are due to some chemical or toxic substance which is inimical to cell- or tissue-integrity. Such substances may be derived from various sources, both within the body and without,—from the vital processes of the body itself, from microbic or vegetable life, from other living bodies or from inanimate nature. Indeed, these products are so numerous, and obstacles to experiment and observation so great, that their extent has not been determined. It is certain, however, that no inflammation can arise without the presence of some specific irritant; and science has gone far enough to demonstrate that, in most cases, such irritant is the product of microörganisms. Senn (Principles of Surgery, page 67,) says: "True inflammation is always caused by the presence of one or more kinds of pathogenic microbes." I do not think this definition is altogether true. Leber (Trans. Oph. Soc. United Kingdom, Vol. XII.), who has most indefatigably studied the causes of inflammation by careful and extended experiment, has proved that certain *chemical* substances absolutely sterilized cause inflammation. He says:

Among the pus-producing chemical substances quicksilver stands in first rank. A single drop, rendered absolutely sterile by heat, antiseptically introduced into the anterior chamber of the eye of an animal, always gives rise to a circumscribed collection of pus in its immediate neighborhood, which, in the course of time, can even lead to purulent infiltration and softening of the inner layers of the cornea. This purulent inflammation is distinguished from that produced by microörganisms by the entire absence of organisms, as can be shown by the usual methods of staining microscopical preparations, and by means of culture experiments made with the purulent exudation of the eye.

He cites copper as another of the commonest chemical substances which produces suppuration in the eye. Croton oil, cantharides, jequrity, arsenic, and many other substances cause inflammation independently of microörganisms. Experiment has also shown that inflammation may be produced by the *products* of microörganisms, the organisms themselves having never been present. Thus Leber (*Ibid*, page 10,) says that "a decoction of the purely cultivated microbes (staphylococcus aurens) in distilled water, in which the microörganisms have been completely killed by heat, still produces an intense purulent inflammation when injected into the anterior chamber (of the eye)." Inflammation, therefore, is not always caused by the presence of microbes, but by the toxines which they produce, as well, and also by chemical products from many other sources.

Whatever substances may cause inflammation, one fact is certain, and that is that the vast majority of inflammations owe their origin to microörganisms and the products which they engender. This being accepted, we must regard inflammations of the eye as essentially microbic in origin, even after traumatism. In some inflammations the special form of microörganism has been demonstrated, as in purulent conjunctivitis. In trachoma the particular microbe is still somewhat conjectural. In all other inflammations of the conjunctiva, and in those of the lachrymal sac, several pathogenic organisms have been found, but without settling definitely what one (or ones) is the specific cause. While, therefore, our present knowledge does not enable us to say just what organisms are the cause of a certain form of inflammation, it is sufficient to permit the general conclusion that microörganisms are the primary offenders, and that our pathology and therapeutics must be shaped to correspond, at least provisionally, till more positive evidence is obtained.

Given, then, a physical and vital condition susceptible to microbic invasion, and all inflammatory diseases of the eye must necessarily be more prevalent and more virulent than in a constitution of strong vital resistance. We may expect, therefore, to find trachoma, and other forms of granular conjunctivitis, in the former much more frequently than in the latter, and our expecta tions are realized. Eczematous eruptions and inflammations of the lachrymal sac and of the margins of the lids are by far more frequent in the former. Inflammations of the cornea occur in the same class oftener than in any other, not to except the syphilitic. Phlyctenular inflammation of the conjunctiva and cornea is scarcely ever present in any but this diathetic class.

The eye and its appendages, in cases of this diathesis, are not alone in being subject to various inflammations. They are in many cases associated with affections of the nose and throat, not in any causal relation, but, coincidentally, phlyctenular disease of the eye, eczema of the lids and orifices of the nose and lip, inflammations of the nasal mucous membrane, adenoid vegetations and enlarged tonsils are often associated; but I do not believe, as Dunn (*New York Medical Journal*, September 19, 1891), Couéteau (*Annales d' Oculistique*, Vol. CVI., 1891, page 401,) and others do, that the phlyctenular disease is caused by the nasal, or naso-pharyngeal disease, or *vice versa*. The constitutional condition simply affords common ground for various bacterial invasions, and different diseases or similar diseases of different structures may consecutively or successively arise, the one not necessarily causing the other. Hence, in treatment the best success cannot be obtained by giving particular attention to any one expression of disease, but to each and every one present, together with such general measures as tend to strengthen tissue-resistance and favor regeneration.

Phlyctenular inflammation of the cornea and conjunctiva, so-called scrofulous ophthalmia, ophthalmia lymphatica, ophthalmia pustulosa, etc., is one of the most common of all diseases of the eyes, and produces more bad vision than any other. It may not cause total blindness as frequently as the purulent forms of conjunctivitis, especially the infantile, but it impairs the vision, even to a serious extent, in far greater numbers.

It is a disease primarily of the epithelial and subepithelial covering of the anterior portion of the globe, or, in other words, of the ocular conjunctiva and anterior layers of the cornea, having a special predilection for the cornea. It is characterized at first by small red elevations, from the size of a millet seed down to minute proportions, which are located at or near the margin of the cornea, sometimes without and sometimes within the margin, and even at the center of the cornea. Sometimes these appear vesicular and sometimes pustular. In a short time the epithelium over these elevations, especially the larger ones, softens or ruptures, and there are then left distinct ulcers. Various conditions, both local and general, and the character of the microbic invasions, shape the future progress of the disease. If tissue-resistance is comparatively strong, and the local environment of the parts is made favorable, the nodule or ulcer heals without further extension. But if, on the other hand, the conditions are reversed, the ulcer may increase in size and depth, or may assume a serpiginous or creeping form; or, instead of becoming an open ulcer, the nodule may become softened without rupturing, and infiltrations of inflammatory products, even of pus, take place, especially in the cornea, to the extent of involving considerable surface. Sometimes the exudation, thus starting superficially in the cornea, extends to the deep layers as a diffused deep-lying infiltration, showing as an opacity of its deeper structure of a uniform grey or yellowish color, becoming fainter towards the edges. In very bad cases these infiltrations break down, forming corneal abscesses, and causing extensive loss of substance. Happily these severe cases, in which an ulcer may extend through the cornea and lead to perforation, or an infiltration may develop into corneal abscess or hypopion, are not common.

The nodules or ulcers may be single or multiple; and if there are more than one, they may be seen in a sort of chain around a portion or all of the margin of the cornea, or they may be separated by considerable space. There are seldom more than three or four ulcers or infiltration-spots when their size is more than that of a mustard seed. In the chain form the elevations are generally minute and do not rupture. After a short time blood-vessels always extend to the phlyctenule, even in the cornea.

The results after healing depend greatly upon the location, extent and depth of the disease-process. If the location has been on the ocular conjunctiva, any cicatricial result is of no consequence. If phlyctenules have appeared superficially on the periphery of the cornea, any loss of transparency, which they may have caused, will not usually affect the vision. But a cicatricial contraction of the outer part of the cornea may so alter the curvature of the central part, as to impair the vision to a large extent. If the central part of the cornea has become the seat of the disease, the vision becomes affected in direct proportion to the extent and depth of tissue involved. A mere destruction of the corneal epithelium causes little or no opacity. But the disease generally reaches deeper than this, and then opacity necessarily and proportionately follows. Of course, the impairment of vision depends more upon the size of the opacity and its extending over the line of vision than upon its density, as a comparatively thin, central opacity reduces vision very much. Fortunately the phlyctenules seldom develop at the exact center of the cornea, although they often encroach upon it. Perforating ulcers and burrowing abscesses of the cornea, when they do occur, are very serious.

Phlyctenular kerato-conjunctivitis is emphatically a disease of childhood and adolescence, and in most cases the general diathesis above referred to is marked. Over-crowding, unhealthful habitations, malnutrition from insufficient or improper diet, etc., increase the susceptibility of individuals to it. I look upon this disease as an eczema of the ball, akin to eczema of the skin. I believe it is dependent upon microörganisms for its origin, although the specific form has not been positively established. The peculiarity of its beginnings and the course which it pursues are exactly what might be expected from such a cause.

The symptoms consist in redness of the eye; development of a fasicular band of blood-vessels running from the sclera toward the elevated, ulcerated or infiltrated spots on the cornea or conjunctiva; much lachrymation, and generally intense photophobia and involuntary spasm of the orbicular muscle of the lids, which are worse when the cornea is involved. This photophobia is accompanied by a peculiar and almost pathognomonic physiognomy of the disease, that of burying the face in the arms or a pillow or cushion.

Exposure of the eye to light frequently causes sneezing. There are sometimes itching and burning of the lids and eyes, but very little pain. Both eyes are quite likely to be affected at the same time, but to this there are many exceptions.

The treatment of this disease resolves itself into improving that general condition which makes its existence possible, and applying such local measures as will destroy its infectious character.

The first indication is medicinally subserved best by giving small doses of arsenic. Tonics, alteratives and "tissue-builders" are generally necessary. Hygienically, regulation of the diet is of paramount importance. It should be plain and nutritious, and regularly served. *All sweets should be positively prohibited.* Other agencies, such as pure air, healthful and cleanly abodes, etc., are not to be forgotten.

Locally some cases require very little, while others demand the greatest skill and closest attention of the practitioner. Instillations of a suturated solution of boracic acid or some other mild collyrium often suffice to thwart the action of the morbific cause. For many decades it has been known, empirically, that calomel dusted into the eyes acted quite effectually in abating this disease. It is now believed that it operates by being converted into bichloride of mercury, thus acting antiseptically. Yellow oxide of mercury ointment has a similar effect. These remedies should be applied daily during the active stages. In obstinate cases I have washed the whole conjunctival and corneal surface with 1 to 1,000 or 1 to 2,000 of bichloride, with most happy results. I have seen cases in children recover immediately after administering ether or chloroform for the purpose alone of facilitating the examination. Hot water, applied for fifteen to twenty minutes over the eyes once or twice a day, has seemed valuable. Instillations of weak solutions of atropia sometimes relieve as if by magic. I do not think, however, that strain or action of the ciliary muscle causes phlyctenules, but it may tend to aggravate the inflammation, and thus atropia becomes a valuable medicament in many cases.

These remedies and others which I might mention of the same class, act beneficially upon the whole conjunctival and corneal surfaces. But, in my opinion, the treatment should not always be restricted to such medication, but the phlyctenules themselves, in whatever stages they may be found, should be destroyed. My plan is to apply the actual cautery to the whole surface, which is elevated, or ulcerated. In this manner the "nidus" of the disease, together with its aggregation of microörganisms, if present, is destroyed, and a comparatively aseptic burn is produced in its stead, which readily heals. This practice has proved so beneficial, that I should feel that I had not done my duty in a severe case, at least, if I had not resorted to it. It is seldom necessary to cauterize a phlyctenular ulcer more than once, although an infiltration may be thus treated several times at intervals of two or three days. The previous treatment suggested is to be continued after the cauterization.

If the patient is old enough and tractable, the different applications can be made, when they are painful, under cocaine anesthesia. When not, chloroform or ether should be administered without hesitation, and I prefer the former, as it affects children kindly.

I will not enlarge upon the treatment of this disease. It should be regarded as an eczema and treated accordingly. I know of no way of preventing its occurrence and recurrence, except by improving the constitutional and environing conditions of children.

To place these upon a plane absolutely beyond the influence of morbific causes is not attainable in our present civilization. But they can be so improved as to make fewer victims, and to lessen society's burden of non-producing dependencies caused by impaired or lost vision.

212 Franklin Street.

